# Thermoregulation is not impaired in breast cancer survivors during moderate‐intensity exercise performed in warm and hot environments

**DOI:** 10.14814/phy2.14968

**Published:** 2021-07-22

**Authors:** Rebecca L. Relf, Ben J. Lee, Gregor Eichhorn, Melanie S. Flint, Louisa Beale, Neil Maxwell

**Affiliations:** ^1^ Environmental Extremes Laboratory University of Brighton Eastbourne UK; ^2^ Occupational and Environmental Physiology Group Coventry University Coventry UK; ^3^ Cancer Stress Laboratory University of Brighton Moulsecoomb UK; ^4^ Centre for Stress and Age‐Related Disease Brighton East Sussex UK

**Keywords:** breast cancer, females, heat reactions, heat stress, inflammation

## Abstract

This study aimed to assess how female breast cancer survivors (BCS) respond physiologically, hematologically, and perceptually to exercise under heat stress compared to females with no history of breast cancer (CON). Twenty‐one females (9 BCS and 12 CON [age; 54 ± 7 years, stature; 167 ± 6 cm, body mass; 68.1 ± 7.62 kg, and body fat; 30.9 ± 3.8%]) completed a warm (25℃, 50% relative humidity, RH) and hot (35℃, 50%RH) trial in a repeated‐measures crossover design. Trials consisted of 30 min of rest, 30 min of walking at 4 metabolic equivalents, and a 6‐minute walk test (6MWT). Physiological measurements (core temperature (*T*_re_), skin temperature (*T*_skin_), heart rate (HR), and sweat analysis) and perceptual rating scales (ratings of perceived exertion, thermal sensation [whole body and localized], and thermal comfort) were taken at 5‐ and 10‐min intervals throughout, respectively. Venous blood samples were taken before and after to assess; IL‐6, IL‐10, CRP, IFN‐γ, and TGF‐β^1^. All physiological markers were higher during the 35 versus 25℃ trial; *T*_re_ (~0.25℃, *p* = 0.002), *T*_skin_ (~3.8℃, *p* < 0.001), HR (~12 beats·min^−1^, *p* = 0.023), and whole‐body sweat rate (~0.4 L·hr^−1^, *p* < 0.001), with no difference observed between groups in either condition (*p* > 0.05). Both groups covered a greater 6MWT distance in 25 versus 35℃ (by ~200 m; *p* = 0.003). Nevertheless, the control group covered more distance than BCS, regardless of environmental temperature (by ~400 m, *p* = 0.03). Thermoregulation was not disadvantaged in BCS compared to controls during moderate‐intensity exercise under heat stress. However, self‐paced exercise performance was reduced for BCS regardless of environmental temperature.

## Graphical abstract

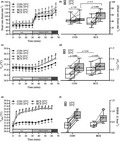

## 1 INTRODUCTION

Breast cancer accounts for 30% of all new cancer diagnoses, equating to one in seven women predicted to develop BC in their lifetime (Bray et al., 2018). Although the prevalence of BC rises each year, there has been a significant reduction in mortality rate in the past two decades, reinforced by survival rates of ~76% in the United Kingdom (Cancer Research UK, 2020) and up to 90% in the United States (Siegel et al., 2019). Regular physical activity has been suggested to reduce cancer recurrence by 21%–35% (Cormie et al., 2017), while also improving cardiorespiratory fitness, and reducing insulin resistance, adiposity, and chronic low‐grade inflammation, alongside an improved quality of life, without adverse side effects (Speck et al., 2010). Increasing physical activity is essential for BCS to begin to offset the reduced peak oxygen consumption (~20%) (Burnett et al., 2013; Jones et al., 2012), increased intermuscular fat (Reding et al., 2019), and prevalence of cardiovascular and/or myocardial injury post‐cancer treatment (Beaudry et al., 2019; Jones et al., 2007), all prognostic of increased risk of cardiovascular disease‐related all‐cause mortality and reduced exercise tolerance. However, survey and epidemiological data estimate that BCS reduce participation in physical activity by ~50% within a year post‐treatment (Littman et al., 2010). Reductions in physical activity are not recovered long term, and post‐treatment side effects are commonly reported as a barrier to exercise in this population, hindering patient's return to normal life (Fong et al., 2012).

An estimated 93% of BCS report at least one adverse side effect 6 months post‐diagnosis (Ellegaard et al., 2017), with symptoms remaining for at least 6 years in 60% of individuals (Schmitz et al., 2012). Negative side effects include, but are not limited to; fatigue, chronic low‐grade inflammation, cardiac toxicity, depression, sleep disturbance, and menopausal/vasomotor symptoms (Carpenter, Elam, et al., 2004; Curigliano et al., 2010; Schmitz et al., 2012). Hot flashes (HF) and the resulting night sweats, indicators of vasomotor symptoms, are two of the most impactful symptoms to physical functioning and quality of life for BCS (Fisher et al., 2013). The prevalence of hot flashes is about 65%, with a third of patients (~30%) reporting them as extremely severe (Carpenter et al., 1998; Chang et al., 2016; Garcia et al., 2015). It is reported that HFs for BCS are more severe and frequent than those associated with natural menopause (Carpenter, Gilchrist, et al., 2004) and although are poorly understood, with majority of the research surrounding menopause rather than BCS, are suggestive of an altered thermoregulatory control (Charkoudian, 2003). First, there is evidence to suggest the thermoneutral zone (between upper threshold for sweating and lower for shivering) is reduced to 0.0℃ in symptomatic women (Freedman & Krell, 1999). On the one hand, this can lead to faster onset of heat dissipation mechanisms (sweating), which is an advantageous thermoregulatory response for those suffering hot flashes. However, the increased sweating response can lead to dehydration more quickly, which can lead to heat‐related illnesses (Coris et al., 2004). Moreover, hot flashes are believed to be triggered by core temperature elevations within this narrowed thermoneutral zone in postmenopausal women (Freedman, 2014; Freedman et al., 1995; Freedman & Woodward, 1996). Currently, there is a paucity of data on the mechanisms underlying hot flashes and thermoregulation in both menopausal women and BCS. It is reported for BCS to experience thermal discomfort from these symptoms (Kokolus et al., 2010), which can be heightened during warm weather and via peripheral heating (Freedman, 1989; Freedman et al., 1992; Kronenberg & Barnard, 1992), therefore warranting further investigation in this population.

Physical activity in hot environments and the associated increase in heat strain, can trigger the release of pro‐ and anti‐inflammatory cytokines (Mündel et al., 2010). These immune alterations may be considered as surrogate markers of the human physiological response to heat stress (Wright et al., 2014). However, to date, previous studies examining cytokine responses to exercise in the heat are sparse and primarily limited to the study of younger (Mündel et al., 2010; Rhind et al., 2004; Selkirk et al., 2008) or older (Wright et al., 2012, 2014) males. Consequently, our understanding of how breast cancer can alter immunological responses to exercise in the heat remain poor, despite a warming climate (Hajat et al., 2014) and rising population of BCS (Bray et al., 2018). Importantly, BCS have been shown to present with a chronic low‐grade inflammatory status (e.g., elevated resting interleukin‐6 and C‐reactive protein) relative to healthy controls. Therefore, it could be plausible that any BCS‐related reductions in thermoregulatory function may be coupled with augmented inflammatory responses relative to age and body size/composition‐matched women following exercise completed in warm and hot conditions. Consequently, more focus is needed on the health and well‐being of BCS and the impacts of physical activity and heat stress have on this population.

The current study aimed to assess how BCS respond in terms of their thermoregulation, inflammatory, and perceptual responses to moderate exercise under heat stress compared to females without cancer, serving as non‐cancer comparators, well matched for age and biophysical characteristics. It was hypothesized that (1) BCS would experience greater thermal strain during moderate exercise under heat stress, (2) BCS would demonstrate a reduced functional capacity in hot conditions, and (3) there would be higher baseline and change post‐exercise in inflammatory markers in BCS compared to controls.

## 2 METHOD

### 2.1 Participants

Twenty‐one (9 BCS (age; 52 ± 7 years, stature; 167 ± 5 cm, body mass; 68.7 ± 8.5 kg, and body fat; 32.1% ± 3.9%), 12 controls [CON] (age; 56 ± 7 years, stature; 167 ± 6 cm, body mass; 67.7 ± 7.3 kg, body fat; 30.0% ± 3.7%)) females were volunteered for this study. All were physically active to the extent that they self‐reported they were able to complete the recommended World Health Organization (WHO) guidelines of physical activity (Bull et al., 2020), this was not assessed via activity monitors. Ethical approval was obtained from the University of Brighton Research Ethics Board (2019‐0365‐Relf), conforming to the principles outlined in the Declaration of Helsinki, 2013, except for registration in a database. All participants provided written and verbal informed consent before any preliminary or experimental trials. Participants were excluded if they had prior or were currently being treated for, respiratory or cardiovascular illnesses or were taking medication that affected thermoregulation. Participants had not experienced hot air temperatures (>25℃) for >3 months, were post‐menopausal, and were non‐smokers. BCS had additional inclusion criteria; females age <65 years, first‐time diagnosis of cancer, no other cancer, considered disease‐free at the time of study enrollment, and at least 4 weeks past completion of the surgery, radiation, or chemotherapy (Table 1). If the women were taking tamoxifen, they were required to have been taking the drug for at least 6 weeks (Carpenter, Elam, et al., 2004). The average quantity and severity (1–10 scale) of hot flashes per day experienced by each BCS were obtained (Table 1).

**TABLE 1:** Details of patients, tumor characteristics, treatment, and hot flash side effects of each BCS, where / indicates no hot flashes experienced

BCS participant	Stage of BC	Year of diagnosis	Type of treatment	Ongoing medication	Date of finished treatment	Years cancer free	Age at diagnosis	Hot flashes (average quantity per/day, severity)
1	Stage 2	Dec‐10	Lumpectomy, mastectomy, chemotherapy, and radiotherapy	Tamoxifen for 6 years	Dec‐11	8	45	5, 6
2	Stage 1	Jan‐18	Surgery	Tamoxifen (10 years), citalopram	Mar‐18	1	47	3, 6
3	Stage 0	Nov‐16	Mastectomy	None.	Feb‐17	2	50	1, 2
4	Stage 3	Oct‐07	Mastectomy, chemotherapy, and radiotherapy	Tamoxifen (10 years), fluoxetine	2008	11	42	6, 7
5	Stage 1, Grade 1	Mar‐18	Mastectomy, chemotherapy, and radiotherapy	Zoladex, Exemestane (10 years)	Feb‐19	<1	38	3, 3
6	Stage 2b, Grade 3 multifocal	2004	Lumpectomy, lymph nodes removed, chemotherapy, and radiotherapy	Citalopram	May‐05	14	40	1, 2
7	Grade 3	Sep‐12	5/16 lymphs removed, mastectomy, 8 chemotherapy sessions, and 15 radiotherapy	Tamoxifen (10 years)	May‐13	6	46	2, 4
8	Stage 2	2010	Radiotherapy (20 sessions of 3 min)	Anastrozole	Jan‐11	8	56	/
9	Stage 2b, Grade 2	2016	Bilateral mastectomy, chemotherapy (10/12/16), and radiotherapy (01/02/17)	Tamoxifen (10 years), tolterodine	Feb‐17	2	52	16, 8

### 2.2 Experimental design

Participants completed one screening session and two experimental blocks, with each block requiring two laboratory visits (five visits in total). During the initial screening session, and after informed consent was provided, stature (Detecto), body mass (Adam Equipment Inc.), and body fat percentage were determined from the measurement of skinfold thickness at four sites (Durnin & Womersley, 1974; Siri, 1956). Thereafter, participants completed a two visit experimental block representative of the mean climate during the UK summer months (both sessions at 25℃, 50% relative humidity [RH]), and a two visit block representative of the peak climate during the UK summer months (both sessions at 35℃, 50% RH) (Waldock et al., 2018). The order of the 25 or 35℃ experimental blocks was randomized via a Latin square design, and all visits were separated by ~7 days. During the first visit of each block (week 1), participants completed a submaximal exercise test (in either 25 or 35℃) to determine the exercise intensity (4 metabolic equivalents [METS]) for the subsequent main experimental trial, which was completed ~7 days later under the same environmental conditions. All participants completed both experimental blocks and all experimental testing was completed in an environmental chamber (TISS).

### 2.3 Standardization of diet and physical activity

Participants were required to abstain from caffeine and alcohol (Shirreffs & Maughan, 2006) 12 h before each session, and to avoid exhaustive exercise (Stewart et al., 2014) for 24 h before trials. Participants were requested to consume similar diets in the 24 h before each trial, with adherence confirmed verbally with participants before each trial (but not formally assessed via diaries). All experimental trials were conducted between 1600 and 1900, to coincide with enhanced viability of sweat output in females (Relf et al., 2019) and the previously identified peak in hot flash occurrence (Freedman, 2014). Participants were required to arrive in a euhydrated state and provide a fresh, mid‐flow urine sample, with hydration status assessed via urine‐specific gravity and urine osmolality following established criteria (Sawka et al., 2007). During each trial, participants wore shorts and a t‐shirt and were not permitted to drink until the final blood sample was drawn.

### 2.4 Submaximal exercise testing

After verbal confirmation that trial standardization had been adhered to, participants were instrumented in a room (~22℃) before completing a 21‐min submaximal test. The test consisted of seven stages, which began at 3.5 km·hr^−1^ and increased every 3 min by 0.5 km·hr^−1^, at 1% gradient (Jones & Doust, 1996) on a treadmill (WoodwayPRO, Woodway GmbH). At 2 min into each stage, ~45 s of expired air was collected using open‐circuit spirometry. Gas samples collected were analyzed using a gas analyzer (Servomex Xentra 4100, Servomex International Ltd, and Buhler Gas Sample Dryer, Type PKE4, Buhler Technologies GmbH). Gas temperature and volume were sampled using a fixed flow pump (model Dymax 30, Charles Austin Pumps Ltd.) and dry gas meter (Harvard Apparatus Ltd). Once analyzed, MET calculation for each stage was acquired and from regression, each equation was used to prescribe the correct intensity for the main trial. After a short rest period, participants were familiarized with the 6‐minute walk test (6MWT; described below) to reduce learning effects (Laskin et al., 2007). This familiarization process was completed in both preliminary trials (25 and 35℃) before each of the main trials.

### 2.5 Main experimental trials

**FIGURE 1:**
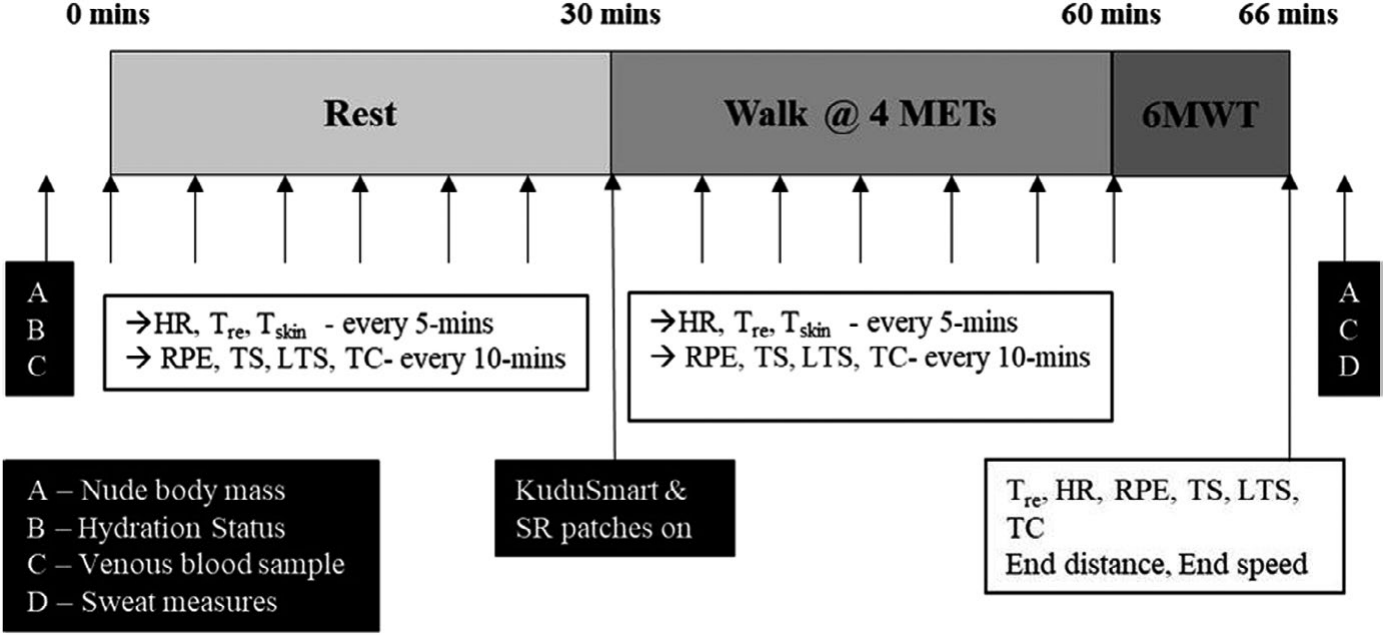
Outline and timings of each of the experimental protocol, where; 6MWT, 6‐minute walk test; HR, heart rate; LTS, local thermal sensation; METs, metabolic equivalents; RPE, ratings of perceived exertion; SR, sweat rate; TC, thermal comfort; *T*_re_, core temperature; TS, thermal sensation; *T*_skin_, mean skin temperature

After verbal confirmation of before‐trial standardization (via verbal questioning), and measurement of hydration status as previously described, nude body mass (NBM) was measured to the nearest 0.01 kg using digital scales (Adam, GFK 150) and participants were instrumented in ambient laboratory conditions (22.3 ± 1.1℃, 32.3 ± 2.5% RH). After instrumentation and 10 min of seated rest, a venous blood sample (~10 ml) was collected from the antecubital fossa. Participants then entered the environmental chamber set to either 25℃/50% RH or 35℃/50% RH and completed a further 30 min of seated rest. At the end of the rest period, participants moved to the treadmill and completed an exercise protocol designed to align with the WHO guidelines of physical activity (Bull et al., 2020) (30‐minute walk at a moderate exercise intensity, 4 METS). Immediately after the 30‐minute walk, functional capacity was assessed via a 6MWT ([Relf et al., 2019; Waldock et al., 2018]; Figure 1), a test shown to be valid and reliable for distance covered on a treadmill (Laskin et al., 2007). When the participant had indicated their readiness to start, the experimenter increased the treadmill speed to 3 km·h^−1^. Participants then self‐reported if they wanted the speed increased or reduced. Core temperature (*T*_re_), heart rate (HR), ratings of perceived exertion (RPE), thermal sensation (TS), local thermal sensation (LTS), thermal comfort (TC), distance covered (m), and end speed (km·hr^−1^) were recorded at the cessation of the 6MWT. All participants were given the same instructions throughout the test.

### 2.6 Instrumentation and measurements

*T*_re_ was monitored throughout all trials using a single‐use rectal probe (449H, Henleys Medical), self‐inserted 10 cm past the anal sphincter. Skin temperature was recorded using skin thermistors (Eltek Ltd) attached to four sites; the midpoint of the right pectoralis major, the midpoint of the right triceps brachii lateral head, right rectus femoris, and right gastrocnemius lateral head. Thermistors were connected to a temperature logger (Squirrel 1000 series, Eltek Ltd.) and mean skin temperature (*T*_skin_) was calculated using the equation by Ramanathan (1964). A HR monitor (Polar FT1, Polar Electro) was affixed to the chest. HR, *T*_skin,_ and *T*_re_ were taken at rest and 5‐min intervals throughout trials, and HR and *T*_re_ were also recorded at the end of the 6MWT. Expired air was collected at three time points during the 30‐minute walk (min 4–5, 14–15, and 24–25) and analyzed as previously described to assess the accuracy of the MET prescription and to calculate metabolic heat production (Cramer & Jay, 2019). Whole‐body sweat rate (WBSR; non‐urine fluid loss) was assessed via NBM taken before and after the trial and reported in L·hr^−1^. Local sweat rate (LSR) was assessed in real‐time using the KuduSmart^®^ wearable monitor and via Tegaderm (Tegaderm™ +Pad, 3 M Health Care) patches, which were placed on the dorsal surface of three sites (lower back, chest, and forearm) and standardized for all participants (Relf et al., 2019).

### 2.7 Subjective ratings

Participants were familiarized with perceptual scales during the submaximal exercise testing visits. RPE (6 = *Very*, *very light* to 20 = *Maximal exertion*) (Borg, 1982; Toner et al., 1986), TS, LTS (0 *Unbearably cold* to +8 *Unbearably hot*) (Toner et al., 1986), and TC (1 = V*ery comfortable* to 6 = *Very uncomfortable*) (Guéritée & Tipton, 2015) were taken at 10‐min intervals during the main experimental trials, and at the end of the 6MWT.

### 2.8 Blood sampling and analysis

Following 10 min of seated rest in ambient temperature before and after the main trials, a 10 ml of venepuncture sample was collected from the antecubital fossa. Blood was transferred into two 5 ml tubes (EDTA Sarstedt, Aktiengesellschaft and Co) and centrifuged (Eppendorf 5702 R Centrifuge) for 10‐min at 4500 revs·min^−1^. Plasma was aliquoted into 1.5 ml microtubes and stored at −86℃ until analysis. Commercially available ELISA kits were used to measure Interleukin‐6 (IL‐6), IL‐10, C‐reactive protein (CRP), Transforming growth factor‐beta (TGF‐β^1^), and Interferon‐gamma (IFN‐γ) in duplicate (Quantikine ELISA kits, R&D Systems). Inter‐plate coefficients of variation were 7% (IL6), 7% (IL10), 10% (CRP), 4% (TGF‐β^1^), and 8% (IFN‐γ). We were unable to gain samples from one participant due to collapsed veins and discomfort from chemotherapy, therefore, *n* = 8 for the BCS group is reported. One participant was below the lower limit of detection (LOD) for resting IL‐10, and another participant was below the LOD for resting IFN‐γ. To account for the missing data points, the lower limit of detection divided by two (LOD/2) was used (LaFleur et al., 2011).

### 2.9 Statistical analyses

Power analysis (G*Power, Version 3.1.5) was conducted using an α‐level of *p* < 0.05, pre‐defined minimal clinically important differences (MCID) between groups for physiological variables, and a moderate effect size (*ƞ_p_* ^2^ = 0.06). A total of 18 participants (9 per group) would result in an 84% probability (i.e., 1−*β*) of detecting a group−condition interaction for rectal temperature (MCID = 0.20℃) and heart rate (MCID = 5 beats·min^−1^), and an 82% probability for detecting a group−condition interaction for whole‐body sweat rate (MCID = 0.2 L·min^−1^). These degrees of change were set due to previous literature determining them as sufficient indicators of heat adaptation (Willmott et al., 2015).

All physiological, perceptual, and inflammatory data were analyzed using jamovi (version 1.6.7). Data are reported as mean ± SD, with alpha set at *p* ≤ 0.05. Before analysis, data were tested for normality and sphericity using the Shapiro–Wilk test. Variables collected at single time points (baseline urine‐specific gravity, urine osmolality, body mass, 6MWT distance, sweat measures, and fold change in cytokines) were compared between the BCS and CON groups using a two‐way mixed‐model analysis of variance (ANOVA) with the factors of temperature repeated (two levels; 25 and 35℃) and group (non‐repeated, BCS and CON). Three‐way mixed‐model ANOVAs with the factors of time (repeated at seven levels for *T*_re_, HR, *T*_skin_; four levels for RPE, TS, LTS, and TC; two levels for inflammatory markers), condition (two levels: 25 and 35℃), and group (two levels: BCS and CON) were employed to evaluate the responses at rest and during and after exercise. When an interaction or main effect was observed, post hoc comparisons were made using the Tukey's HSD test. For all significant main effects and interactions, effect sizes were calculated as partial eta squared (*ƞ_p_* ^2^) to provide an objective indication of the magnitude of difference. For reference, values of 0.01, 0.09, and 0.25 are considered small, medium, and large effects (Lakens, 2013).

## 3 RESULTS

### 3.1 Participant characteristics and equality of study conditions

Mean participant characteristics, hydration status, exercise intensity, metabolic heat production, and ambient conditions are presented in Table 2. The CON and BCS groups were not different in age, stature, body mass, body surface area, or body fat percentage. Both groups were similarly hydrated before each main trial, as indicated by osmolality and urine‐specific gravity values. There were no differences in exercise intensity, metabolic heat production, or environmental conditions noted between groups or conditions (Table 2).

**TABLE 2:** Baseline standardization of physical anthropometrics, activity, and temperatures prior to the 25 and 35℃ conditions for BCS (*n* = 9) and CON (*n* = 12)

Category	Group	Variable	25°C	35°C	*p* value, *ƞ_p_* ^2^	
					Condition	Interaction
Urine	BCS	Osmolality (mOsm·kg^−1^)	149 ± 99	138 ± 86	0.648, 0.011	0.513, 0.023
	CON		232 ± 169	293 ± 228		
	BCS	Urine‐specific gravity	1.003 ± 0.003	1.008 ± 0.16	0.327, 0.050	0.485, 0.026
	CON		1.006 ± 0.006	1.007 ± 0.007		
Exercise intensity	BCS	Average METs	4.01 ± 0.33	4.16 ± 0.29	0.855, 0.002	0.444, 0.031
	CON		4.16 ± 0.29	4.10 ± 0.28		
${\dot{H}}_{\text{prod}}$	BCS	Absolute (W)	251 ± 31	258 ± 31	0.107, 0.131	0.606, 0.014
	CON		248 ± 32	252 ± 24		
	BCS	Relative (W·kg^−1^)	3.71 ± 0.24	3.81 ± 0.13	0.096, 0.139	0.744, 0.006
	CON		3.67 ± 0.17	3.75 ± 0.19		
Main trial conditions	BCS	Temperature (°C)	25.05 ± 0.12	35.22 ± 0.23	<0.001, 0.999	0.765, 0.005
	CON		25.03 ± 0.14	35.17 ± 0.22		
	BCS	Humidity (%)	50.81 ± 0.81	49.61 ± 1.02	0.073, 0.159	0.462, 0.029
	CON		51.6 ± 1.26	51.08 ± 2.31		

Category	Variable	BCS	CON	*p* value
Anthropometrics	Age (years)	52 ± 7	56 ± 7	0.437
	Stature (cm)	167 ± 5	167 ± 6	0.438
	Body mass (kg)	68.70 ± 8.50	67.73 ± 7.25	0.910
	Body surface area	1.77 ± 0.12	1.76 ± 0.11	0.953
	Body fat (%)	32.1 ± 3.9	30.0 ± 3.7	0.074
Oxygen consumption	Average VO2 (ml·kg^−1^·min^−1^)	10.04 ± 1.58	9.21 ± 1.53	0.236

Data are shown as mean ± SD. Exercise intensity and ${\dot{H}}_{\text{prod}}$ were calculated from an average of three gas samples taken over the 30 min of exercise.

### 3.2 Cardiovascular responses

**FIGURE 2:**
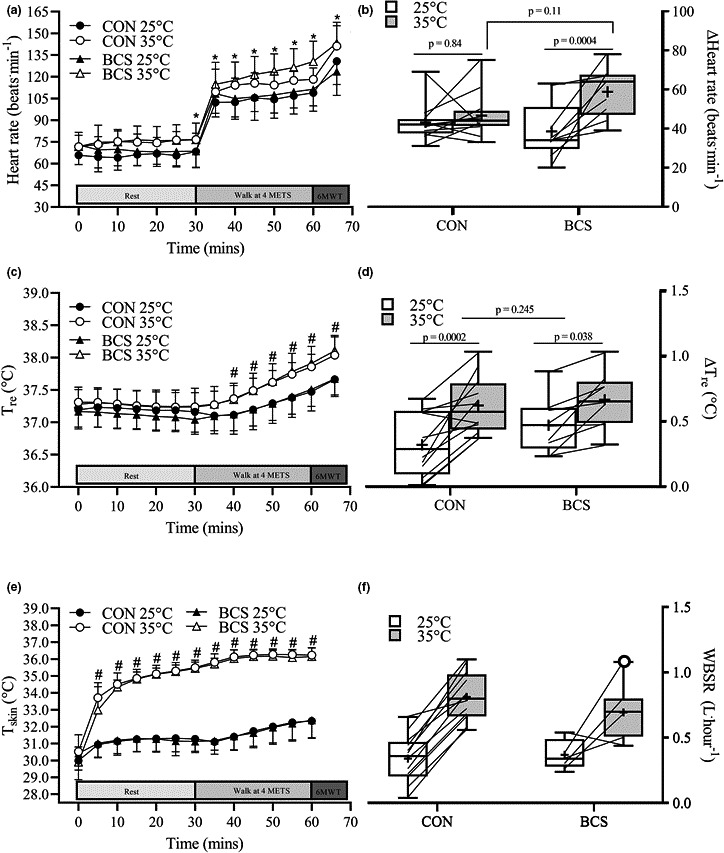
HR (a, b), *T*_rec_ (c, d), and *T*_skin_ (e) responses of CONT and BCS throughout rest, 30 min of exercise, and following the 6MWT in 25 and 35℃ conditions. Panels on the left display the mean and SD at each time point, and panels on the right display each change (lines) from rest to the end of the 30‐min exercise bout. Box plots display the median (mid‐line), mean (cross), and the 25 and 75th interquartile ranges (boxes), and whiskers illustrate the highest and lowest values. Panel (f) displays WBSR responses for the CONT and BCS groups after exercise in the 25℃ (white boxes) and 35℃ (gray boxes) conditions. Where; *different from rest (*p* < 0.05), ^#^different between 25 and 35℃ (*p* < 0.05), ^ǂ^different between BCS and CON (*p* < 0.05)

Prior to the start of the 4 MET walk, resting HR was elevated by ~8 beats·min^−1^ in 35℃ compared to 25℃ (effect of condition, *p* < 0.0001, *ƞ_p_* ^2^ = 0.92), though there was no condition × group interaction (*p* = 0.23, *ƞ_p_* ^2^ = 0.07; Figure 2a). Thereafter, HR responses exhibited a time × condition interaction (*p* = 0.023, *ƞ_p_* ^2^ = 0.12) whereby HR was elevated by ~12 beats·min^−1^ at each time point throughout the 35℃ condition compared to 25℃. When expressed as the change in HR from the first resting measurement to the end of exercise, a main effect for condition (*p* = 0.0019, *ƞ_p_* ^2^ = 0.41), and a condition × group interaction was found (*p* = 0.02, *ƞ_p_* ^2^ = 0.25; Figure 2b). Post hoc analysis revealed that the change in HR was not different between the 25 and 35℃ conditions for CON (~4 beats·min^−1^ difference; *p* = 0.84). In contrast, a ~20 beats·min^−1^ difference was observed between the 25 and 35℃ conditions in the BCS group (*p* = 0.004), equating to a ~12 beats·min^−1^ difference from CON. However, after correction for multiple comparisons, the difference between groups was not statistically different (*p* = 0.11).

### 3.3 Thermoregulation

There was a main effect for condition (*p* = 0.00008, *ƞ_p_* ^2^ = 0.57), time (*p* < 0.00001, *ƞ_p_* ^2^ = 0.871), and a condition × time interaction (*p* < 0.00001, *ƞ_p_* ^2^ = 0.556) for rectal temperature (Figure 2c). Post hoc analysis revealed that *T*_re_ was not elevated during the 30‐min rest period for either condition, and was therefore similar prior to the start of the 4 MET walk for both CON (25℃: 37.16 ± 0.31℃, 35℃: 37.24 ± 0.22℃) and BCS (25℃: 37.04 ± 0.22℃, 35℃: 37.25 ± 0.28℃; condition × group interaction: *p* = 0.822). During exercise in the 35℃ condition, *T*_re_ was elevated from rest from 10 min of exercise onwards (*p* < 0.001), and from 20 min onwards in the 25℃ condition (*p* < 0.0001). Rectal temperature was higher in the 35℃ versus 25℃ condition after 10 min of exercise and remained ~0.3–0.4℃ higher at each time point. A similar response was observed between CON and BCS groups (condition × time × group interaction, *p* = 0.19). When expressed as change from rest, there was a main effect for condition (*p* = 0.00002, *ƞ_p_* ^2^ = 0.627), such that the change in *T*_re_ was ~0.25℃ greater in the 35℃ compared to 25℃ condition (*p* = 0.0002), with this change being similar between the CON and BCS groups (condition by group interaction, *p* = 0.245; Figure 2d).

There was a main effect for condition (*p* < 0.00001, *ƞ_p_* ^2^ = 0.967), time (*p* < 0.00001, *ƞ_p_* ^2^ = 0.820), and a condition × time interaction (*p* < 0.00001, *ƞ_p_* ^2^ = 0.433) for *T*_skin_ (Figure 2e). Post hoc analysis revealed that *T*_skin_ was elevated from 5 min of rest in the 35℃ condition and throughout each remaining time point, with no difference observed between CON and BCS groups (condition × time × group interaction, *p* = 0.952).

WBSR was ~0.4 L·hr^−1^ greater after the 35℃ trial compared to the 25℃ trial (main effect of condition, *p* < 0.00001, *ƞ_p_* ^2^ = 0.837; Figure 2f). Although the condition × group interaction did not meet conventional levels of statistical significance (*p* = 0.084, *ƞ_p_* ^2^ = 0.149), visual inspection of the data revealed that one BCS participant recorded a WBSR ~0.40 L·min^−1^ greater than the group mean after the 35℃ condition (1.1 vs. 0.70 L·hr^−1^ BCS group mean, clear circle in Figure 2f).

All measurements of localized sweat rate were elevated in the 35℃ trials relative to the 25℃ trial (main effects for condition all *p* < 0.05, Table 3), and no differences between the CON or BCS groups were found (condition × group interaction all *p* > 0.05).

**TABLE 3:** Whole‐body sweat rate and local sweat rate measures during the 25 and 35℃ conditions for BCS (*n* = 9) and CON (*n* =  12)

	25℃ trial		35℃ trial		Main effects	
	BCS	CON	BCS	CON	Condition	Group
WBSR (L·hr^−1^)	0.37 ± 0.11	0.34 ± 0.18	0.70 ± 0.20	0.81 ± 0.18	<0.00001	0.51
LSR (mg·min^−1^·cm^−2^)						
Back	0.23 ± 0.16	0.25 ± 0.19	1.08 ± 0.61	1.31 ± 0.41	<0.00001	0.35*
Chest	0.14 ± 0.08	0.12 ± 0.08	1.16 ± 1.04	1.16 ± 0.55	0.00001	0.96*
Upper arm	0.06 ± 0.05	0.05 ± 0.04	0.25 ± 0.14	0.27 ± 0.13	<0.00001	0.83
Forearm	0.07 ± 0.08	0.08 ± 0.08	0.35 ± 0.14	0.46 ± 0.18	<0.00002	0.19
KuduSmart	0.44 ± 0.20	0.38 ± 0.09	1.27 ± 0.30	1.33 ± 0.32	<0.00003	0.98*

Data are shown as mean ± SD.

* Indicates non‐parametric statistics.

### 3.4 Functional performance

**FIGURE 3:**
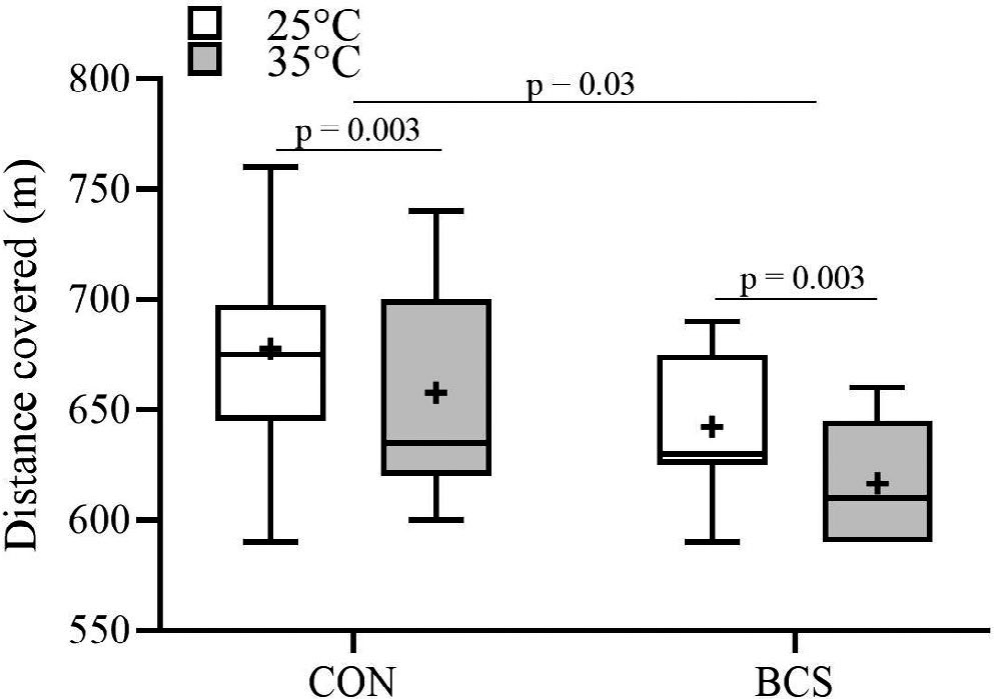
Mean and individual participant data points between BCS (*n* = 9) and CON (*n* = 12) for the end distance in the 6MWT for both temperature conditions. Box plots display the median (mid‐line), mean (cross), and the 25 and 75th interquartile ranges (boxes), and whiskers illustrate the highest and lowest values. Lines represent each individual participant performance in the 25℃ (white boxes) and 35℃ (gray boxes) conditions

There was a main effect for condition (*p* < 0.0036, *ƞ_p_* ^2^ = 0.377) and group (*p* = 0.036, *ƞ_p_* ^2^ = 0.210), but no condition × group interaction (*p* = 0.683, *ƞ_p_* ^2^ = 0.009) for distance covered in the 6MWT. Both groups covered a greater distance in the 25℃ versus 35℃ (by ~200 m; *p* = 0.003). In addition, the control group covered more distance than BCS, regardless of environmental temperature (by ~400 m, *p* = 0.03; Figure 3).

### 3.5 Perceptual responses

RPE, TC, TS, and LTS all increased throughout the exercise (main effect of time, *p* < 0.001, *ƞ_p_* ^2^ = 0.802), and were higher in the 35℃ versus 25℃ conditions (main effect condition) for both experimental groups (group−condition × time interaction, *p* < 0.001, *ƞ_p_* ^2^ = 0.823), as demonstrated in Table 4.

**TABLE 4:** Perceptual scores for BCS (*n* = 9) and CON (*n* = 12) during the 25 and 35℃ conditions Data are shown as mean ± SD

		25℃					35℃					*p* value, *ƞ_p_* ^2^		
		0	10	20	30	Post−6MWT	0	10	20	30	Post‐6MWT	Condition	Time	Interaction
TS	BCS	4 ± 0	5 ± 0	5 ± 1	5 ± 1	6 ± 1	5 ± 1	6 ± 1	6 ± 1	7 ± 1	7 ± 1	0.753, 0.005	0.209, 0.076	0.839, 0.015
	CON	4 ± 1	5 ± 1	5 ± 1	5 ± 1	6 ± 1	4 ± 1	5 ± 1	6 ± 1	7 ± 1	7 ± 1			
LTS	BCS	4 ± 0	5 ± 0	5 ± 1	6 ± 1	7 ± 1	5 ± 1	6 ± 1	6 ± 1	7 ± 1	8 ± 1	0.369, 0.043	0.392, 0.051	0.024, 0.152
	CON	4 ± 0	5 ± 1	5 ± 1	5 ± 1	6 ± 1	5 ± 1	6 ± 1	6 ± 1	6 ± 1	7 ± 1			
TC	BCS	1 ± 1	3 ± 1	3 ± 1	3 ± 1	4 ± 1	4 ± 1	4 ± 1	5 ± 1	5 ± 1	6 ± 1	0.745, 0.006	0.105, 0.101	0.370, 0.053
	CON	2 ± 1	3 ± 1	3 ± 1	3 ± 1	4 ± 1	3 ± 1	4 ± 1	4 ± 1	5 ± 1	5 ± 1			
RPE	BCS	6 ± 0	10 ± 2	11 ± 2	12 ± 2	14 ± 2	6 ± 0	11 ± 3	13 ± 3	13 ± 3	15 ± 3	0.349, 0.046	0.417, 0.048	0.294, 0.063
	CON	6 ± 0	11 ± 1	12 ± 1	12 ± 1	14 ± 2	6 ± 1	12 ± 1	12 ± 1	13 ± 2	15 ± 2			

### 3.6 Inflammatory responses

Absolute concentrations for all inflammatory measurements, the ANOVA interaction, and effect size are presented in Table 5. Resting cytokine concentrations were not different between groups (all *p* > 0.20). Expressed as a change from rest, IL‐6 was increased by ~0.75 pg·ml^−1^ after exercise (main effect of time, *p* = 0.0002, *ƞ_p_* ^2^ = 0.536), regardless of condition (main effect of condition = 0.194) or group (*p* = 0.96). In contrast, IL‐10 was unchanged after exercise in the 25℃ condition, but increased by ~1.8 pg·ml^−1^ after exercise in the 35℃ condition (main effect of condition, *p* = 0.034, *ƞ_p_* ^2^ = 0.226), and was similar between groups (*p* = 0.82). The resulting IL6/IL10 ratio was higher after exercise in 25 versus 35℃ (by ~0.6; main effect for condition, *p* = 0.016, *ƞ_p_* ^2^ = 0.279), which was not different between groups (*p* = 0.86). IFN‐γ concentrations increased by ~0.82 pg·ml^−1^ after exercise (main effect of time, *p* = 0.035, *ƞ_p_* ^2^ = 0.235), regardless of condition (main effect of condition, *p* = 0.327) or group (*p* = 0.794). CRP was increased after exercise in both the 25℃ (by ~0.37 mg·L^−1^) and 35℃ (by ~0.29 mg·L^−1^) condition for the BCS, and only increased following the 35℃ condition in CON (by ~0.14 mg·L^−1^). After exercise, CRP was ~0.39 mg·L^−1^ higher in the BCS versus CON (group effect, *p* = 0.0002) regardless of condition. TGF‐β^1^ was unchanged from rest in either condition (*p* = 0.525) and was not shown to be different between groups (*p* = 0.459).

**TABLE 5:** Inflammatory responses before and after 25 and 35℃ conditions between BCS (*n* = 8) and controls (*n* = 12)

		25℃			35℃			*p* value, *ƞ_p_* ^2^		
		Pre	Post	Change	Pre	Post	Change	Condition	Time	Interaction
IL‐6 (pg·ml^−1^)	BCS	1.15 ± 0.43	1.85 ± 0.46*	0.70 ± 0.55*	3.02 ± 1.71	4.31 ± 1.77*	1.29 ± 1.77*	0.00009, 0.582	0.00024, 0.536	0.506, 0.025
	CON	1.58 ± 1.60	2.27 ± 1.21*	0.69 ± 0.66*	2.90 ± 1.31	4.24 ± 1.84*	1.34 ± 1.94*			
IL‐10 (pg·ml^−1^)	BCS	3.03 ± 1.88	2.92 ± 2.63	0.11 ± 1.23	2.84 ± 1.12	4.12 ± 1.90*	1.28 ± 2.13*	0.148, 0.112	0.286, 0.063	0.834, 0.002
	CON	7.03 ± 8.30	6.77 ± 9.92	0.26 ± 2.55	6.56 ± 6.81	8.58 ± 11.24*	2.02 ± 5.27*			
IL‐6/IL‐10 ratio	BCS	0.53 ± 0.35	1.60 ± 1.43**#**	1.07 ± 1.37**#**	1.11 ± 0.56	1.26 ± 0.70	0.15 ± 0.76	0.267, 0.068	0.015, 0.283	0.583, 0.017
	CON	0.35 ± 0.33	1.03 ± 1.12**#**	0.67 ± 1.09**#**	0.83 ± 0.68	1.23 ± 0.93	0.39 ± 0.98			
CRP (mg·L^−1^)	BCS	1.53 ± 1.27	1.90 ± 1.26*ǂ	0.37 ± 0.39ǂ	1.34 ± 0.71	1.63 ± 1.01*	0.30 ± 0.41ǂ	0.397, 0.040	0.004, 0.370	0.562, 0.019
	CON	1.71 ± 1.07	1.45 ± 0.92	0.26 ± 0.59	1.47 ± 1.08	1.61 ± 1.28*	0.15 ± 0.33			
TGF‐β^1^ (pg·ml^−1^)	BCS	13.74 ± 3.64	13.96 ± 2.13	0.23 ± 2.89	10.91 ± 3.44	11.95 ± 4.30	1.05 ± 3.19	0.268, 0.068	0.016, 0.283	0.583, 0.017
	CON	16.10 ± 6.56	16.50 ± 7.08	0.40 ± 2.49	12.45 ± 7.19	13.29 ± 5.80	0.85 ± 2.29			
IFN‐γ (pg·ml^−1^)	BCS	4.77 ± 3.02	5.75 ± 3.31*	0.98 ± 1.64*	2.85 ± 1.82	3.59 ± 2.42*	0.74 ± 1.45*	0.00005, 0.632	0.035, 0.235	0.518, 0.025
	CON	4.50 ± 4.95	5.51 ± 4.33*	1.01 ± 2.55*	2.51 ± 3.90	2.86 ± 3.80*	0.35 ± 084*			

Data are shown as mean ± SD. Where; * different from rest (*p* < 0.05), ^#^different between 25 and 35 (*p* < 0.05), ^ǂ^different between BCS and CON (*p* < 0.05).

## 4 DISCUSSION

This study aimed to examine whether breast cancer survivors differ in thermoregulatory, perceptual, and inflammatory responses at rest, during, and after moderate exercise in warm (25℃) and hot (35℃) conditions compared to a carefully matched control population. The principal findings of this study are: (1) when exercising according to the current WHO‐prescribed guidelines (30 min of moderate exercise; ~4 METS) (Bull et al., 2020), thermoregulatory function, perceptions of effort, thermal comfort, and thermal sensation were similar between age‐matched controls and BCS in both warm and hot conditions; (2) functional exercise performance assessed, using a validated 6‐minute walk test, was impaired in BCS regardless of the temperature the test was completed in; (3) of the markers of systemic inflammation measured, only CRP was shown to be different between experimental groups and was ~0.33 mg·L^−1^ higher after exercise in BCS versus controls, regardless of environmental temperature, which is not enough to alter risk category for cardiovascular disease in adults (Pearson et al., 2003). No differences at rest or following exercise were noted for IL‐6, IL‐10, IFN‐γ, and TGF‐β^1^. Overall, the data from this study provides evidence that thermoregulation, perceptions of temperature sensation/comfort, and the after‐exercise inflammatory responses in warm and hot conditions are not compromised in BCS when compared to control participants well matched for biophysical characteristics. Despite no differences in physiological or perceptual responses versus healthy controls, BCS self‐selected a lower work intensity during the functional capacity 6MWT which led to reduced distances regardless of environmental condition and is suggestive of some degree of functional impairment.

To the best of our knowledge, this is the first study that examines the thermoregulatory responses of BCS during both warm and hot environmental conditions. We incorporated an exercise duration and intensity derived from the current WHO guidelines for physical activity and previous research in our laboratory for vulnerable populations (Waldock et al., 2018), which allowed us to investigate the physiological responses to this novel cohort under conditions they could be realistically expected to experience throughout the summer months. Importantly, our control and BCS groups were carefully matched for age and biophysical characteristics––key methodological considerations that can reduce potential bias when exploring thermoregulation between two independent groups (Cramer & Jay, 2015). We aimed to recruit BCS that self‐reported symptoms that are indicative of vasomotor impairments, with 8/9 of our cohort reporting regular hot flashes (compared to 1/12 in the control group). By ensuring our BCS experienced hot flashes, we ensured our experiment would be able to explore the anticipated reduced/altered thermoneutral zone (Freedman, 2014) in BCS compared to matched controls.

Our original hypothesis, that BCS who suffer from symptoms indicative of impaired vasomotor function (e.g., hot flashes and night sweats), may also display impaired thermoregulation, is not supported by our data. Both experimental groups exercised at the same relative and absolute rate of metabolic heat production during each condition (~3.7 W·kg^−1^/~250 W), and consequently, we observed similar *T*_re_ responses throughout rest and during exercise between groups (Figure 2c,d). We acknowledge that changes in body heat content are underestimated when determined by thermometry (e.g., changes in rectal temperature) when compared to direct calorimetry, thus any reduction in the ability of BCS to dissipate heat may not always be reflected in the measurements of core body temperature. Although we ensured a constant thermal drive (i.e., a metabolic heat production of 3.7 W·kg^−1^, Table 2) in both experimental cohorts, the levels of heat strain and acute nature of our study may not have been of sufficient intensity or duration to identify differences in thermoregulatory responses between groups, with moderate ∆*T*_re_ of 0.3–0.5℃ in 25℃ and 0.6–0.7℃ in 35℃ observed. We completed all experimental testing between 1600 and 1900 h–a period previously associated with the natural circadian peak for hot flashes in menopausal women (Carpenter et al., 2002, 2012). However, no participant in either group reported any symptoms before, during, or after the experimental trials. It is plausible that the occurrence of hot flashes may display a different circadian rhythm in those recovering from breast cancer compared to menopausal women (Carpenter et al., 2002; Freedman, 2014). Our data suggest that BCS can adhere to present physical activity guidelines without the risk of increased negative side effects associated with impaired vasomotor responsiveness. Our study specifically addresses a brief (~70 min) acute exposure––responses over consecutive days or more prolonged exposures to heat stress, such as those encountered during a heatwave event requires further research.

Heart rate was assessed to examine the interactive effect of environmental temperature and disease state on the cardiovascular strain. When expressed as a change from resting HR, the control population shows greater cardiac stability, with only ~10 beats·min^−1^ (range: −10 ± 23 beats·min^−1^) difference between the end of exercise at 25℃ and the commensurate time point at 35℃. In contrast, a ~20 beats·min^−1^ (range: 7 ± 46 beats·min^−1^) difference between 25 and 35℃ conditions was observed for BCS (Figure 2b). Although the interaction did not reach conventional levels of statistical significance (*p* = 0.11), potential between‐group differences in cardiovascular responses warrant some brief consideration as the difference between conditions is ~4‐fold higher than our pre‐determined minimum clinically important difference (5 beats·min^−1^). During exercise in the heat, HR is increased in line with skin temperature to support the greater skin blood flow requirement that attends a narrowing core‐skin thermal gradient. Given that both core and skin temperatures were not different between groups, and that both dry and evaporative heat loss responses were also similar between groups, it is unlikely that ~12 beats·min^−1^ difference in final HR observed between the 35℃ condition can be attributed solely to thermoregulatory impairments. One potential explanation is a clinically significant cardiac dysfunction induced after exposure to anthracyclines, a class of chemotherapeutics (Curigliano et al., 2010). In the present study, participants who underwent treatment with anthracyclines (*n* = 6) had heart rates of 5–10 beats·min^−1^ higher at all time points in the 25℃ condition, and ~7–10 beats·min^−1^ higher at all time points in 35℃ condition compared to BCS who did not undergo this treatment (*n* = 3), demonstrating a degree of cardiac instability. Further emphasized by absolute HR’s indicating greater cardiovascular strain post 30‐min exercise between groups in 35℃ (BCS vs. CON; 131 ± 14 vs. 119 ± 8 beats·min^−1^). We employed a widespread non‐categorizing approach during recruitment, allowing all types of BCS to be enrolled in the study. As a result, our sample includes an array of; treatment types, medication, length post‐treatment, and diagnosis, and so we cannot make firm inferences regarding treatment types and effects to physiological function, and particularly the effects of anthracyclines on cardiac function. Research specifically investigating thermal/physiological responses in BCS treated with anthracyclines and/or other treatment modalities is warranted because it is feasible that these variations may impact physiological responses.

Interestingly, BCS self‐selected a lower work intensity during both environmental temperatures compared to controls, and as a consequent completed ~400 m less distance, irrespective of similar aerobic fitness observed in preliminary tests (Table 2). This is less than the error for a treadmill 6MWT for current data (typical error of measurement: 2.1%–4.6% [13–29 m] between groups), and previous literature in varying populations (5.2% [23 m] (Olper et al., 2011) and when performed over‐ground (4.2% [16.6 m] (Sandberg et al., 2020). The reduction in physical performance appears to have occurred independently from physiological cues (e.g., skin temperature) known to influence pacing behaviors (Schlader et al., 2011; Tucker et al., 2006). Consistent with our physiological observations, no between‐group differences for perceptions of effort were observed in either environmental condition during the 30‐min steady‐state walking. Despite some evidence that BCS experienced elevated cardiovascular strain during 35℃, both groups displayed similar RPE values (13 ± 3 and 13 ± 2), equating to a verbal queue of “somewhat hard.” To establish if perceptions of thermal sensation had been affected due to breast cancer diagnosis and treatment, we examined localized thermal sensation to the chest region (LTS). LTS values followed an identical pattern to overall TS, rising throughout the exercise, and higher in 35 versus 25℃. No differences were noted for eccrine sweating, specific to the chest region (Table 3), suggesting that the local sweat rate in this specific body region (chest) and thermal sensation is not affected after BC treatment. We are unable to provide a clear reason for the reduction in functional performance, but suggest that this could also be related to treatment‐induced impairments in cardiovascular function in a subset of our BCS population.

Associations between small, prolonged increases in plasma inflammatory cytokines and chemokines, and persistent fatigue have been reported in cancer survivors (Bower & Lamkin, 2013)––a population already characterized by reductions in physical activity levels (Littman et al., 2010). Although there is some evidence which suggests inflammatory markers are elevated in BCS when compared to healthy women (Seruga et al., 2008), many studies are not well controlled for lifestyle factors known to influence the measurement of systemic cytokines (e.g., pre‐sampling physical activity levels, no or poorly reported dietary control, lack of control for circadian variation, and different sampling times), and are also confounded by clinical factors which alter systemic inflammatory measurements, limiting interpretation. In the present study, we use two experimental groups, well matched for both age and physical characteristics, and implemented well‐controlled pre‐trial standardization protocols regarding diet, physical activity, and circadian variation. We show that both the control and BCS groups had resting IL‐6 concentrations (~1.5–4.5 pg·ml^−1^) well within the healthy range reported for middle‐aged adults (Ridker et al., 2000). Resting IL‐6 in BCS was similar to concentrations reported in the Yale Exercise and Survivorship study (~1.9–3.6 pg·ml^−1^) (Jones et al., 2013) and far below concentrations previously reported in some BCS cohorts (19.7 ± 41.2 pg·ml^−1)^ (Rogers et al., 2013) and 16.3 ± 25.9 pg·ml^−1^ (Gómez et al., 2011). Resting IL‐10, IFN‐ γ, TGF‐β^1^, and CRP were also similar between our experimental groups at rest, and reflective of normative concentrations reported in the wider literature for older populations, regardless of disease state (Kim et al., 2011). Discrepancies between studies are difficult to reconcile and could reflect simple population differences; differences in body composition–with increased adiposity known to elevate inflammatory markers (Pudkasam et al., 2017); a range of years after diagnosis; and type and sensitivity of assays utilized–with older multiplex assays known to be less sensitive than ELISAs available at the time (Gómez et al., 2011).

In the present study, the similar thermal, cardiovascular and perceptual strain responses observed between the controls and BCS were paralleled by similar between‐group cytokine responses in the period immediately after exercise, regardless of environmental temperature. Given that the participants in the current study were physically active in daily living (although not trained), and physical exercise programs have been shown to reduce inflammatory responses in both healthy populations and those recovering from BC (Meneses‐Echavez et al.,; Mills, 2017; Petersen & Pedersen, 2005), it would be of interest to examine the systemic cytokine responses to exercise in warm and hot conditions in more sedentary or less healthy BCS, and make comparisons to similarly well‐matched controls. It remains unknown whether more prolonged or repeated exercise periods in the heat exacerbates the inflammation or cytokine response and symptoms associated with such disorders, potentially putting less healthy BCS at increased risk of heat‐related injuries, illnesses, or health complications during prolonged heat wave events.

### 4.1 Perspectives

Breast cancer survivors report negative side effects (e.g., fatigue and hot flashes) and lack knowledge and confidence regarding safety of physical activity (Pekmezi et al., 2012), all of which are possible contributors to the reported 30%–47% of the BCS population not achieving the recommended daily activity guidelines (Casla et al., 2014). A chronic low‐grade inflammatory state has also been suggested to impact physical activity behaviors and sensations of fatigue and is also known to alter thermoregulatory control. Although speculative, we suggest that BCS may have self‐selected a slower pace as a form of anticipatory behavioral thermoregulation based upon previous experiences of vasomotor symptoms (HFs), acting pre‐emptively to ensure excessive heat accumulation does not occur via a reduction in exercise intensity before any sensory physiological signals (e.g., increased *T*_re_) are initiated (Tucker et al., 2006).

Our study provides evidence that healthy BCS can achieve 30 min of moderate exercise guidelines in both warm and hot conditions, without negative consequences or side effects usually attributed to this condition, and with no alterations in inflammatory status when compared to females with no history of breast cancer. The results of this study represent a starting point, in which further work investigating thermoregulatory and cardiovascular function during exposures with durations and intensities that are representative of those experienced during heatwaves is required. Establishing whether the results presented herein are applicable to physical activity performed across multiple days (i.e., 3 days per week physical activity guidelines, or more prolonged heatwave simulations) would enable the development of bespoke physical activity guidelines for this specific population and which can be tailored toward reducing the negative consequences and side effects of both exercise and high environmental temperatures.

### 4.2 Considerations

The present study was conducted at one exercise intensity and duration designed to reflect the current guidelines for daily physical activity. It is plausible that if the duration of exercise or the length of exposure to environmental heat had been longer, alterations in physiological function, or subjective reporting of symptoms such as hot flashes would become apparent. The population of BCS in the present study self‐report as adhering to current physical activity guidelines, and likely represent healthy members of the BCS community, therefore, may mitigate any potential detrimental side effects. This is reinforced by BCS covering a greater distance than healthy individuals, of similar age, in previous literature (~300 m more) (Sperandio et al., 2015). Work in which less active and healthy BCS with chronic low‐grade inflammation undergo similar protocols is, therefore, necessary in order to better characterize the overall population, and determine whether guidelines tailored toward mitigating health risks associated with exposure to heat need to be targeted/adjusted for different members of the BCS community.

For methodological limitations, the MCIDs were established from heat adaptation data in a healthy population (Willmott et al., 2015). It is acknowledged that utilizing these data may not hold the same validity as if from a clinical population; however, in the absence of bespoke clinical data in BCS, it was utilized as a foundation to establish meaningful differences between groups. Moreover, while the induction of hot flashes was not the focus of this paper, it would have been interesting and valuable to measure vasomotor function directly, therefore, future research should endeavor to include this in their design.

## 5 CONCLUSION

No compromise in any physiological, inflammatory, or perceptual parameters was observed between BCS and controls before, during, and after exercise in both warm and hot conditions. BCS self‐selected a reduced work intensity during a test of functional capacity (6MWT) in both warm and hot conditions, without differences in measured physiological or perceptual markers. However, a heightened cardiovascular strain was highlighted in the data between populations and therefore may explain the reduced capacity to work. Further investigations into the impact of BC on the cardiovascular strain, especially those post‐chemotherapy, are therefore warranted.

## CONFLICT OF INTEREST

The authors have no conflict of interest to disclose. The results of the present study do not constitute endorsement by ACSM. The results of the study are presented clearly, honestly, and without fabrication, falsification, or inappropriate data manipulation.

## AUTHOR CONTRIBUTION

RR, NM, MF, and LB conceived the study. RR and NM developed the experimental design. RR and GE conducted the data collection. RR and BL conducted all statistical analysis. All authors reviewed the final manuscript.
